# Implementing virtual reality based surgical topographic anatomy for the education of medical students: a pilot study

**DOI:** 10.1186/s12909-025-08563-z

**Published:** 2026-01-09

**Authors:** Jan Philipp Ramspott, Alexander D. Bungert, Carsten Szardenings, Isabelle Flammang, Felix Becker, Markus Holling, Felicia Kneifel, Andreas Pascher, Haluk Morgül

**Affiliations:** 1 Department of General, Visceral and Transplant Surgery, University Hospital Münster, Albert-Schweitzer-Campus 1, Building W1, Waldeyerstraße 1,, Münster, 48149 Germany; 2 Institute of Biostatistics and Clinical Research, University of Münster, Münster, 48149 Germany; 3 Department of Neurosurgery, University Hospital Münster, Münster, 48149 Germany

**Keywords:** Surgery, Teaching, Questionnaire, Extended reality, VR

## Abstract

**Background:**

Surgical specialties necessitate a comprehensive understanding of anatomy. Traditional two-dimensional anatomical teaching methods lack the depth required for spatial comprehension. Virtual reality (VR)-based anatomy models offer a promising alternative, enabling an enhanced understanding of surgical topographic anatomy. This study investigated the feasibility and teaching value of VR-based surgical anatomy teaching. In addition, we explored the impact of pursuing a future surgical career.

**Methods:**

Meta Quest 2 VR system with Medicalholodeck “Anatomy and Dissection Master XR” software applications was used for virtual segmentation of a mediastinal and abdominal model annotated with anatomical terminology. The feasibility, knowledge gain, and teaching value of the VR-based topographic surgical anatomy were evaluated using a newly developed 14-item self-report questionnaire that included numeric rating scales (0–10; 0: no knowledge gain/teaching value; 10: high knowledge gain/teaching value) and dichotomous questions.

**Results:**

Thirty-seven medical students performed the instructor-guided virtual segmentation. Participants reported a high knowledge gain (median 8) and perceived teaching value (median 9) regarding complex mediastinal anatomy. Four students (11%) reconsidered their later professions after the VR sessions. Statistical analyses did not reveal any significant differences among students of different ages, genders, or educational levels.

**Conclusions:**

This study showed that the implementation of VR-based surgical topographic anatomy teaching for medical student education is feasible. As an additional tool to conventional anatomical teaching, it could enhance teaching value irrespective of age, gender, or educational level, and may influence subsequent surgical career decisions. The questionnaire requires further refinement including the standardized assessment of anatomical knowledge.

**Supplementary Information:**

The online version contains supplementary material available at 10.1186/s12909-025-08563-z.

## Background

Surgery necessitates a comprehensive understanding of topographic anatomy and its complex spatial organization and relationships with adjacent structures. Although anatomical teaching commences early in medical studies, deficiencies in anatomical knowledge have been consistently reported among medical students and surgical residents [1–5]. To date, anatomical teaching has predominantly relied on traditional teaching methods, including two-dimensional (2D) forms of delivery, such as anatomical images [6]. Three-dimensional (3D) teaching, such as cadaveric dissection enables a deeper understanding of anatomy with its spatial structures from numerous viewpoints, but is constrained by certain safety, financial, accessibility limitations, and ethical dilemmas. Furthermore, the COVID-19 pandemic has underlined the compelling necessity of innovative digital 3D teaching methods [7–10].

Virtual reality (VR)-based anatomy models have demonstrated potential as an effective tool for enhancing the anatomical knowledge of both medical students and surgical residents [11–13]. Despite this potential, the application of VR in the teaching of surgical topographic anatomy has not yet undergone extensive evaluation or been systematically integrated into medical education curricula. This may be attributed to factors related to accessibility, cost-effectiveness, and the existing knowledge gap concerning a standardized evaluation of VR teaching effectiveness [12, 14].

It is of pivotal importance to further investigate the feasibility of implementing VR-based surgical anatomy teaching for medical students and the associated learning outcomes. Therefore, our study aimed to further explore VR-based surgical mediastinal and abdominal anatomy teaching exemplified by a virtual esophageal cancer patient. These anatomical areas were selected because they represent central and key anatomical regions in general surgery, where complex spatial relationships are critical for surgical planning and intervention. In addition, we examined the impact of VR on medical students’ interest in surgery, and subsequently, on their future surgical career choices.

## Methods

### Study cohort

A total of 37 medical students, who participated in our voluntary VR program at the University Hospital of Münster, Department of General, Visceral, and Transplant Surgery, between November 2022 and May 2024, were included in the study. All participating students had prior anatomical education (anatomical lectures and cadaveric dissection) in the first two years of medical school before taking part in the study. Written informed consent was obtained from all participants at the beginning of the study.

### VR software and hardware

**Fig. 1:**
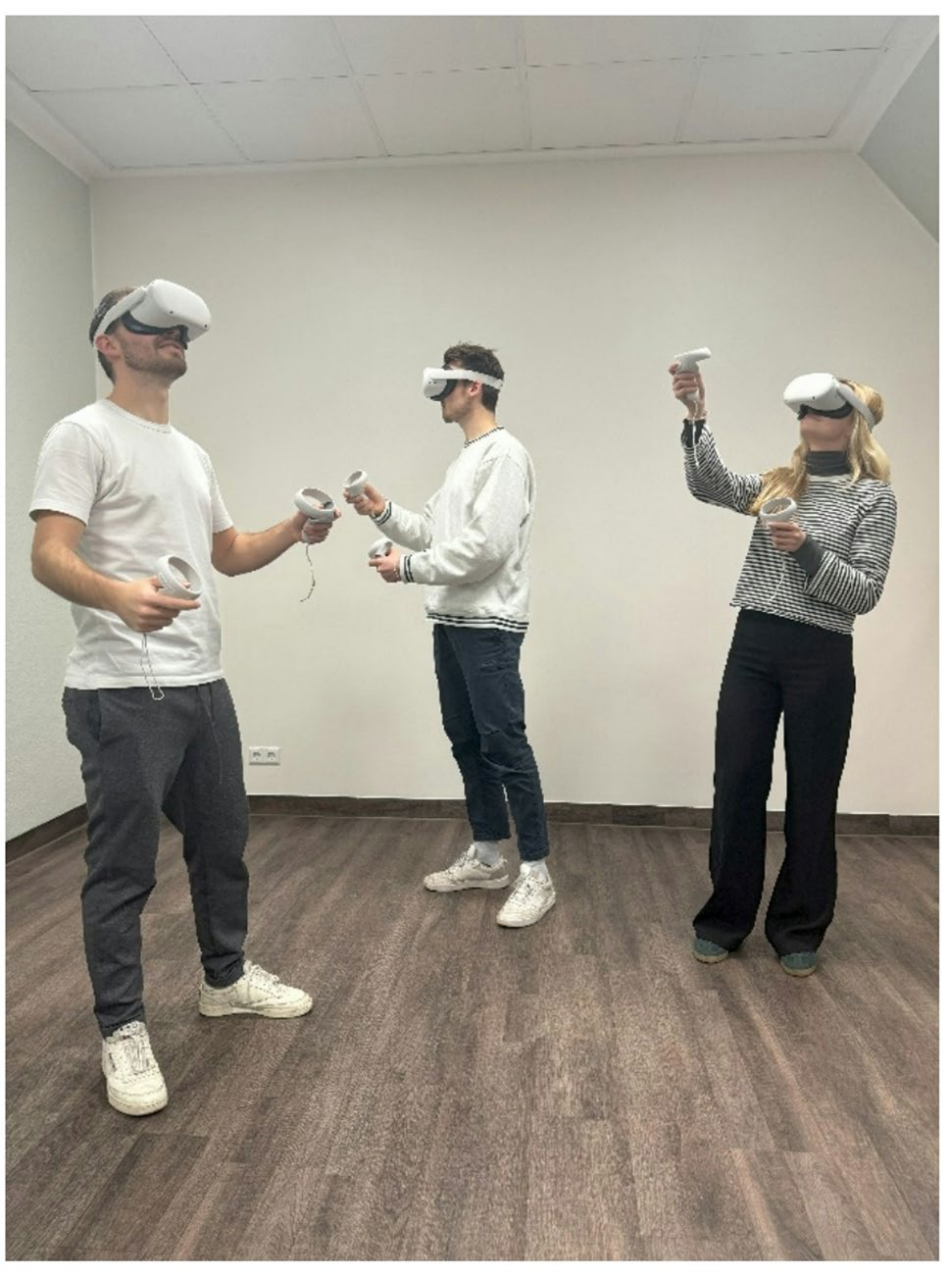
During the VR session, students used the Meta Quest 2 virtual reality (VR) headset and wrist and touch motion controllers for virtual segmentation

Meta Quest 2 VR system (Meta, California, USA) was used for virtual segmentation of an upper and lower body model incorporating all mediastinal and abdominal anatomical structures. Each student wore a VR headset and two wrist and touch motion controllers to interact with the model (Fig. 1). Software applications (Anatomy Master and Dissection Master XR) were provided by Medicalholodeck (Switzerland). The Anatomy Master software facilitated the examination and virtual segmentation of schematic 3D anatomical mediastinal and abdominal structures, whereas the Dissection Master provided 3D cadaveric anatomical mediastinal and anatomical structures. The VR software allowed users to select and drag different anatomical structures. In addition, the software provided information regarding the selected anatomical structures. All students were connected online via a cloud-based application to ensure consistent conditions for all study participants. All students and teaching staff interacted and communicated via worn headsets within the same metaverse environment.

### Ethics statement

According to Sect. 15 [of the “Medical Association of Westphalia-Lippe” professional code of conduct, a duty to consult only exists for biomedical research projects on humans, not for a pure teaching evaluation. Therefore, ethical approval is not required (reference number: 2021-237-f-N). Clinical trial number: not applicable.

### Study procedure

The VR program was conducted over two consecutive days between November 2022 and May 2024, with each session lasting 90 min. The student groups comprised two to five students. The topic of the VR session was not disclosed to students beforehand.

On the first day, an experienced surgical resident and/or surgical consultant (instructor) introduced the hardware setup and software interface through a guided demonstration via a shared cloud interface. After the initial walkthrough, students were given the opportunity to individually explore the virtual environment and basic interaction tools (e.g., object manipulation, navigation, virtual segmentation, and annotation features) under the instructor’s supervision. These activities were designed to build technical confidence, ensure smooth participation, and to minimize cognitive load during the subsequent learning task following the next day.

On the second day, the instructor presented a virtual clinical case using a case-based learning approach. *A 50-year-old male patient presented to his primary care physician with dysphagia that had persisted for several months. Furthermore*,* the patient reported unintentional weight loss and night sweats.*

An esophageal cancer case was selected because of its association with mediastinal and abdominal anatomies spanning different complex anatomical regions. Following the case presentation, students were instructed to obtain additional information regarding the patient’s medical history. Subsequently, they were requested to propose potential diagnoses and to recommend further diagnostic steps. Utilizing various interactive software interfaces, the study participants initially performed a virtual segmentation of the anatomical model with the aim of obtaining a 3D view of the entire mediastinal and abdominal areas. This individual exploration phase was followed by a collaborative cloud-based session. Here, all participants, including the instructor, were connected within a shared virtual environment via a secure cloud system.

**Fig. 2:**
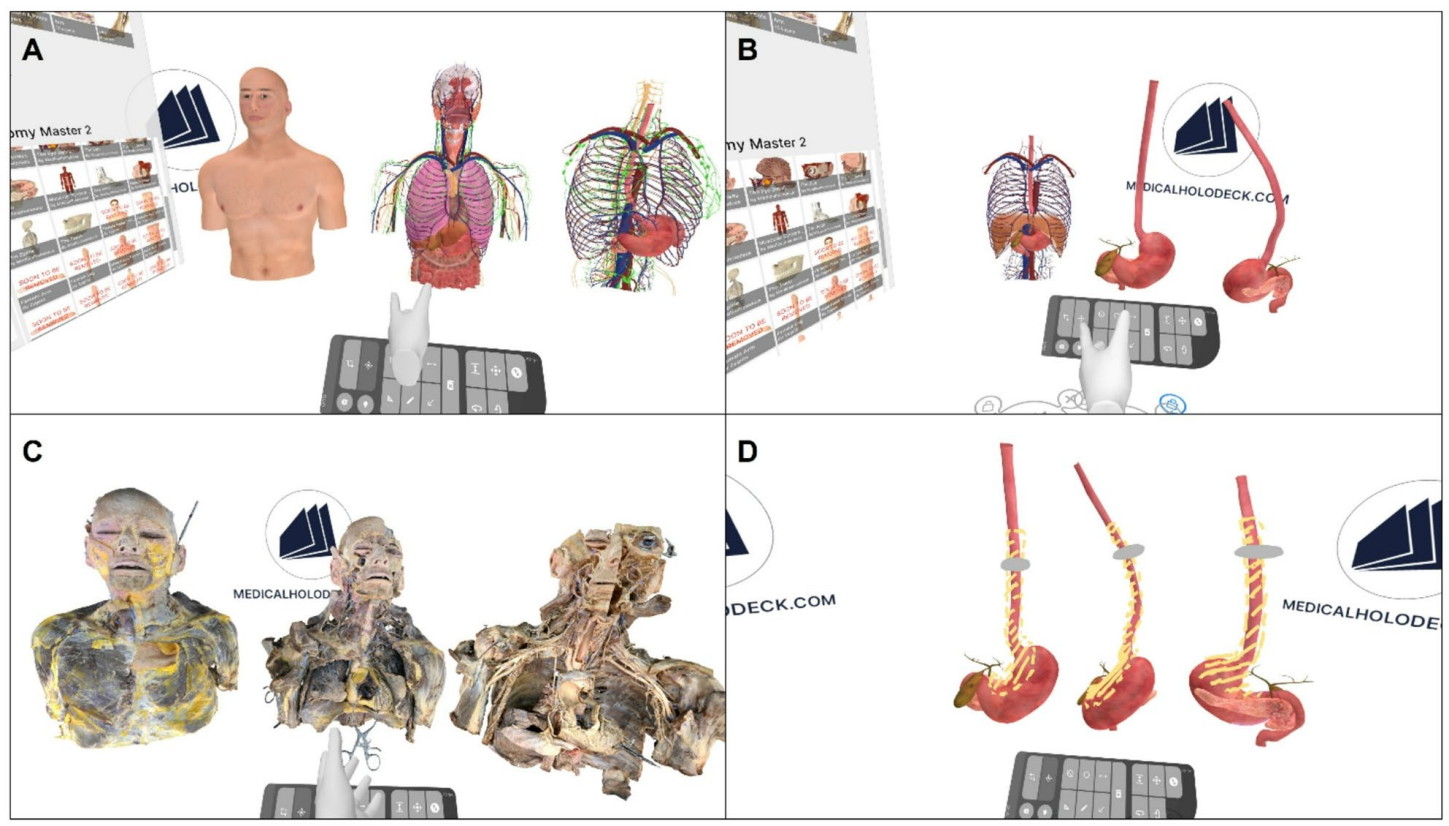
Screenshots of the virtual reality (VR)-based segmentation procedure from students’ perspective (**A**-**D**). The toolbar for the operation and rotation of the 3D model is shown below. Schematic anatomical models (Medicalholodeck, Anatomy Master) (**A**, **B**), cadaveric anatomical models (Medicalholodeck, Dissection Master) (**C**), and esophagectomy procedure with gastric conduit reconstruction including marked resection boundaries (yellow) and tumor (grey) are shown (**D**)

The virtual segmentation was performed using controllers that simulated different selected tools for the manipulation and rotation of the 3D model. Initially, the anatomical model (Anatomy Master) and subsequently, the cadaveric anatomical structures (Dissection Master) were dissected (Fig. 2A/B and Fig. 2C, respectively). Students were supposed to identify mediastinal organ position, arterial, venous, lymphatic, and nerve pathways, as well as the esophageal course and length.

The cloud-based phase enabled the instructor to observe each student’s virtual field of view, verify anatomical identifications in real time, provide corrections if necessary, and demonstrate optimal navigation pathways.

Following virtual segmentation, the instructor conducted a virtual schematic demonstration of the esophagectomy procedure and gastric conduit reconstruction, including delineation of resection boundaries based on the minimally invasive approach (Fig. 2D). Again, all participants and the instructor were connected through the shared cloud-based virtual environment, allowing simultaneous participation and real-time visualization of the instructor-led esophagectomy demonstration.

### Questionnaire

After completing the virtual segmentation procedure, all participants were asked to complete an anonymous paper-based 14-item questionnaire (quasi-voluntary survey) without any time restrictions. No survey incentives were offered, and data were collected as a single measure collection. Single questionnaires were directly distributed to each participant to avoid redundant responses. The questionnaire included demographic data questions. Furthermore, it included several questions asking students to rate the feasibility and self-reported teaching value of VR-based topographic surgical anatomy teaching. Regarding the usefulness, usability, knowledge gain, and teaching value of the VR exercises, the ratings were based on a discrete numerical scale ranging from 0 (meaning useless/difficult to use/nothing) to 10 (meaning very useful/very workable/very much). On this scale, a value of five was designated as neutral. Additional opinions were provided on the questionnaire. Anonymity and confidentiality of all study participants were maintained throughout the study period. The survey questions are detailed in Supplementary Fig. 1.

### Statistical analyses

All statistical analyses were performed using the program R version 4.4.1 [15]. Responses to categorical and dichotomous questions were summarized as absolute (counts) and relative frequencies (percentages). The ratings (usefulness, usability, knowledge gain, and teaching value) were summarized by their median and interquartile range, as well as their mean for completeness.

The structure of the seven non-demographic questions was evaluated via an exploratory maximum-likelihood factor analysis and the internal consistency of any (sub)scales consistent with the results of the factor analysis was determined using Cronbach’s alpha.

All graphs were created using GraphPad Prism 10.4.0 (GraphPad Software, Massachusetts, USA). For comparison of subpopulations differing by level of education, gender, and age, Fisher’s exact test was used for dichotomous and categorical responses, and the Mann-Whitney-U Test for ratings, as the assumption of interval scale level for ratings was not presumed. The local significance level for *p*-values was set at *p* ≤ 0.05.

## Results

### Study cohort

The baseline characteristics of the students are shown in Table 1. The study included 37 medical students from both last- and non-last year training levels. The study predominantly comprised female students (*n* = 27, 73%). The median age was 25 years. The majority of the participants were right-handed (*n* = 35, 95%). Twenty-one students (57%) were in their last year of medical studies, and only half of the study cohort reported prior VR experience (*n* = 19). Moreover, 51% (*n* = 19) of the students indicated that they had already determined their future medical professions. Among these, 35% (*n* = 13) specified that a surgical specialty was the primary choice.

**Table 1:** Baseline characteristics of study cohort

	*N* (%)
Gender	
Male	10 (27.0)
Female	27 (73.0)
Median age (range), years	25 (20–30)
Handedness	
Right	35 (94.6)
Left	1 (2.7)
n/a	1 (2.7)
Level of training	
Last year	21 (56.8)
Non last year	16 (43.2)
Prior virtual reality experience	
Yes	19 (51.3)
No	18 (48.7)
Are you already sure about your later profession?	
Yes	19 (51.3)
Surgical profession	13 (35.1)
Non-surgical profession	5 (13.5)
n/a	1 (2.7)
No	18 (48.7)

### Evaluation of the questionnaire

The factor analysis suggested a subscale consisting of the four rating scale items as a measure of “perceived personal benefit” of the VR exercises. The unweighted sum score of these items had a Cronbach alpha of 0.80 (CI 0.67–0.89) indicating a high internal consistency. The factor analysis is detailed in Supplementary Tables 1 and Supplementary Fig. 2.

### General perception of VR exercises

**Fig. 3:**
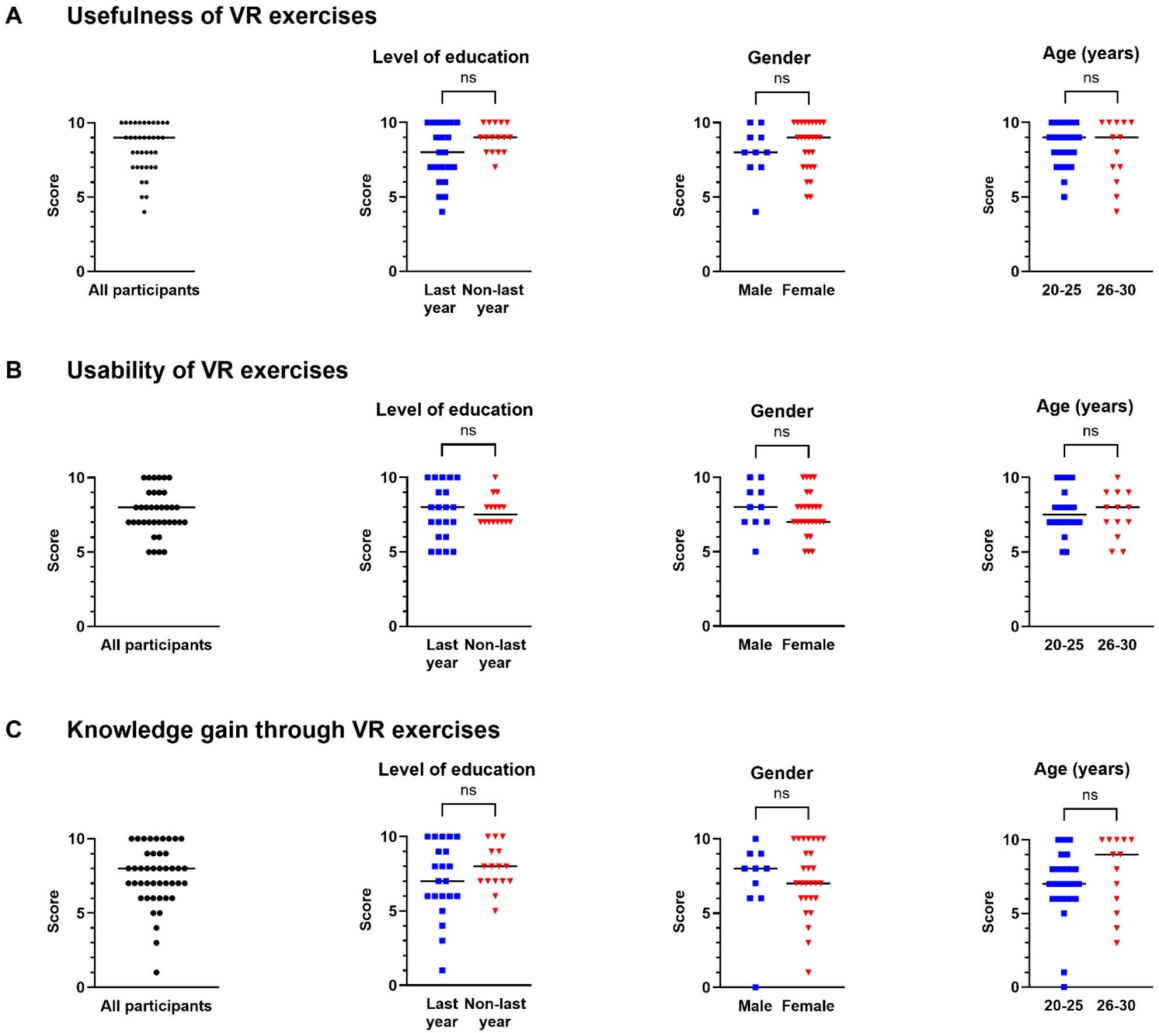
General perception of virtual reality (VR) exercises (**A**: usefulness, **B**: usability, **C**: knowledge gain). The ratings were based on a discrete numerical scale ranging from 0 (useless/difficult to use/no knowledge gain) to 10 (very useful/very workable/high knowledge gain). Each data point represents one participant. The median is indicated by the horizontal line. Data were analyzed using the Mann-Whitney-U Test. VR: virtual reality, ns: not significant

Upon completion of the virtual segmentation procedure, all the participants were asked to complete an anonymous 14-item questionnaire. The response rate was 100%. All survey questions addressing VR application and general knowledge gain revealed high scores for each category (Fig. 3). All participants reported high perceived usefulness of the VR exercises, with a median usefulness score of 9 on a discrete numerical rating scale ranging from 0 (useless) to 10 (very useful). Statistical analyses did not reveal any significant differences between the different levels of education (last vs. non-last year students), gender (male vs. female students), or age groups (20–25 years vs. 26–30 years old students). However, non-last year and female students tended to rate usefulness higher (Fig. 3A).

VR usability assessment revealed a median score of 8 among all participants on a discrete numerical rating scale ranging from 0 (difficult to use) to 10 (very workable). No significant differences were observed across the levels of education, genders, or age. Only male and older students (26–30 years) tended to have higher usability ratings (Fig. 3B).

When asked about the acquisition of new learning content using the VR setting, all participants reported high knowledge gain (median score 8 [scale from 0, indicating no knowledge gain, to 10, signifying high knowledge gain]). Last-year, younger (20–25 years), and female students provided comparatively lower ratings than non-last year, older (26–30 years), and male students, although these differences did not reach statistical significance (Fig. 3C).

### Teaching value of VR exercises

**Fig. 4:**
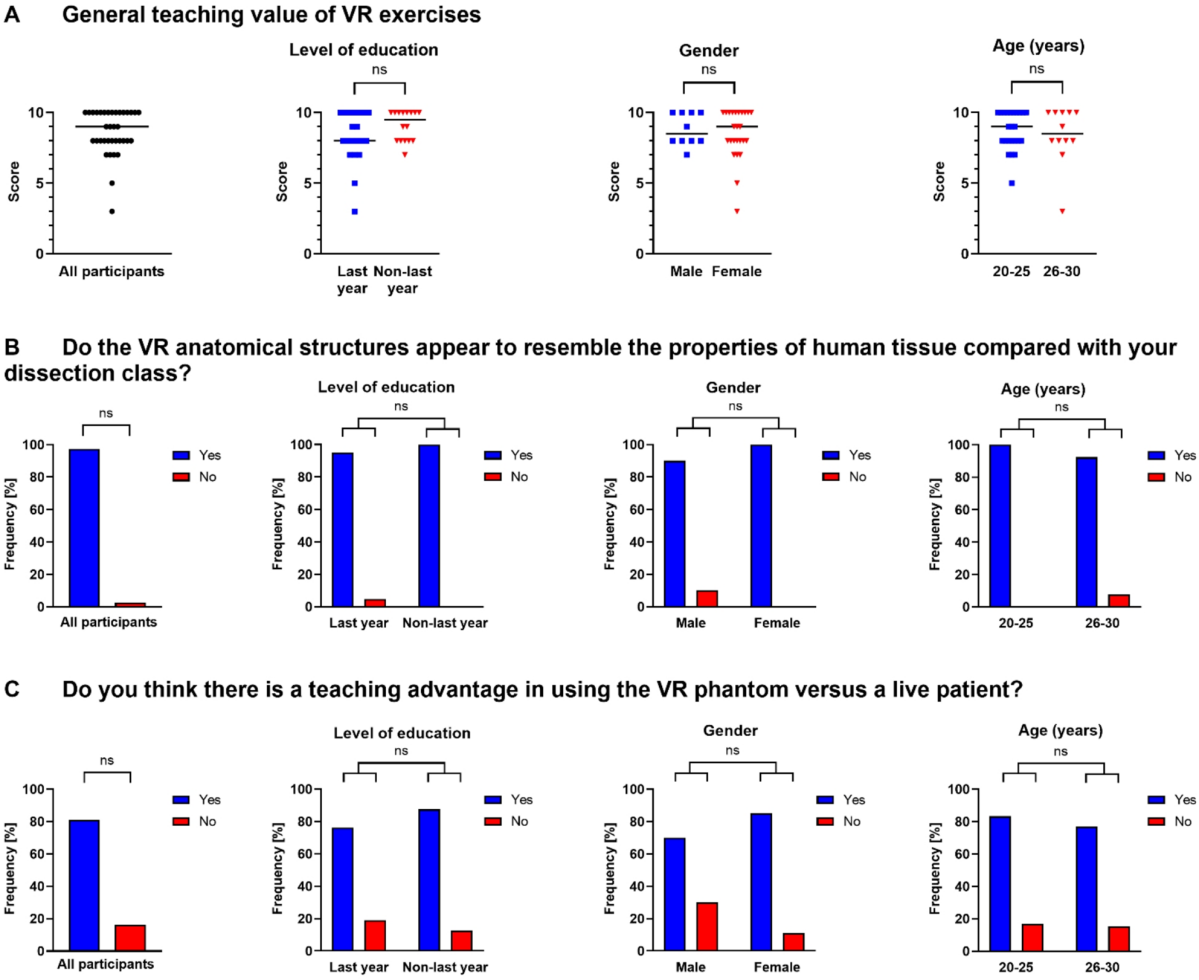
Teaching value of virtual reality (VR) exercises (**A**: general teaching value, **B**: resemblance, **C**: teaching advantage) . The ratings were based on a discrete numerical scale ranging from 0 (no added teaching value) to 10 (high added teaching value). Each data point represents one participant. The median is indicated by a horizontal line. Relative frequencies (percentages) of the observed responses are shown. Data were analyzed using the Mann-Whitney-U test for ratings and Fisher’s exact test for dichotomous and categorical responses. VR: virtual reality, ns: not significant

The second objective of the survey was to estimate the teaching value of the VR applications. In general, the study participants attested a high perceived teaching value. Among all the students, the general median teaching value score was 9 on a discrete numerical rating scale ranging from 0 (no added teaching value) to 10 (high added teaching value). Statistical analyses did not reveal any significant differences in perceived added teaching value between last and non-last year, male and female, or younger (20–25 years) and older (26–30 years) students. Non-last year medical students showed a tendency towards higher teaching value scores (Fig. 4A).

Next, students were asked about the resemblance of the VR-simulated anatomical structures to their dissection class, including cadaveric anatomical models, based on dichotomous questions (yes/no). The vast majority (97%) of participants affirmed this question. Comparing the different levels of education (last year vs. non-last year students), gender (male vs. female students), and age (20–25 vs. 26–30 years old students), no statistically significant differences were observed (Fig. 4B).

Based on this query, the study participants were also asked about the teaching advantages of a virtual patient compared to a live patient. A total of 81% of students (*n* = 30/37) affirmed the teaching advantage of the virtual patient. Four last year students (19%) and 12% non-last year students (*n* = 2) did not perceive a teaching advantage for VR patients. Furthermore, fewer male students (70%, *n* = 7) tended to have a VR teaching advantage than female students (85%, *n* = 23). No statistically significant differences were observed between the groups. One participant did not respond to this question (Fig. 4C).

### Personal view of study participants, role of instructor, choice of later profession

**Fig. 5:**
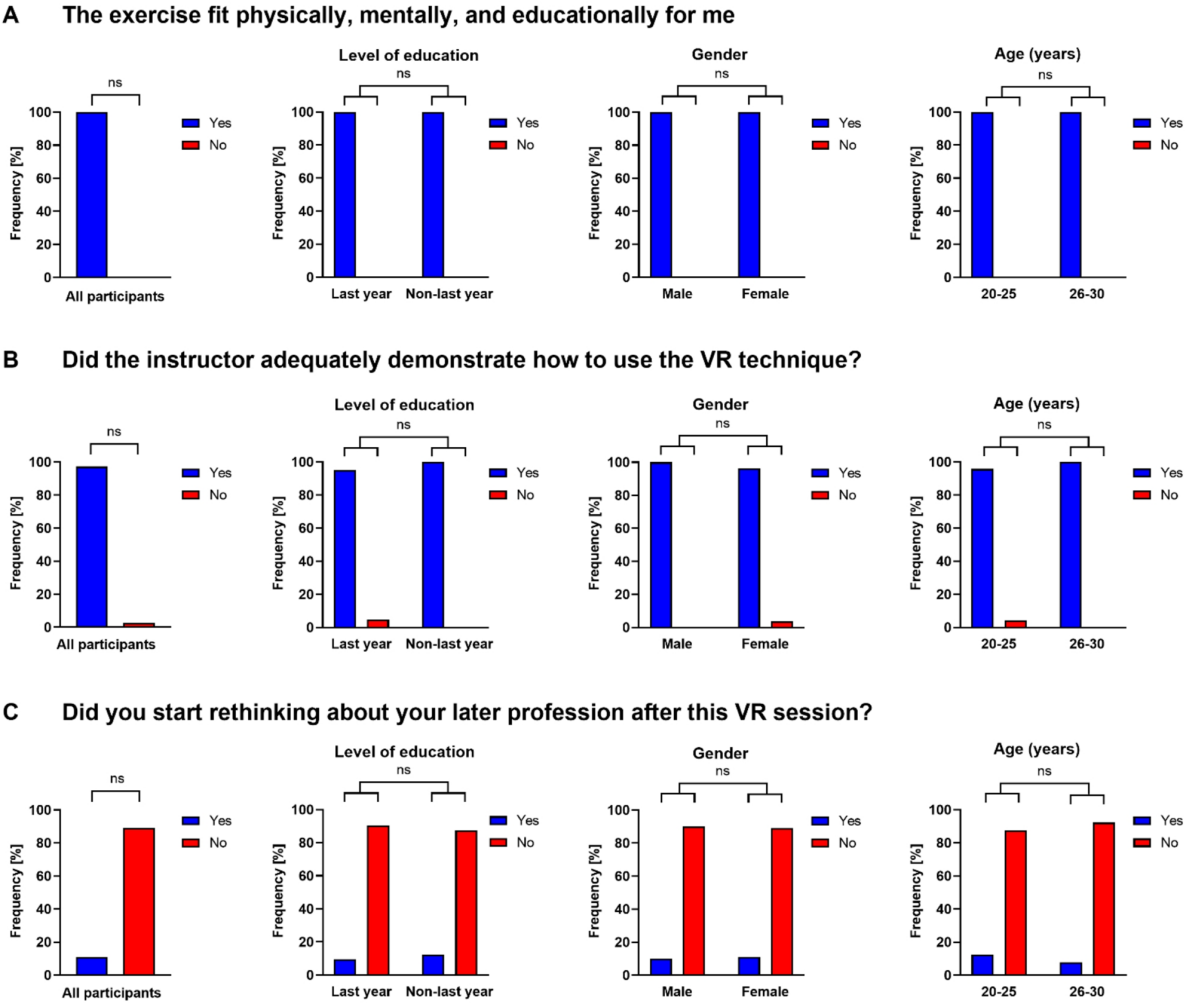
Personal view of study participants (**A**), role of instructor (**B**), and choice of profession after virtual reality (VR) exercises (**C**). Data were analyzed using Fisher’s exact test and are shown as the relative frequencies (percentages) of the observed responses. VR: virtual reality, ns: not significant

The third focus of the survey included questions regarding the participants’ personal views, including their subsequent professional choices, and the role of the instructor. All study participants indicated that the VR exercises were suitable both physically and mentally. Statistical analyses did not reveal any significant differences between last and non-last year, male and female, or younger (20–25 years) and older (26–30 years) students concerning the personal physical or mental value of the VR exercises (Fig. 5A).

Being asked for the role of the instructor, only one student reported that the instructor did not adequately demonstrate the use of the VR technique (Fig. 5B).

Finally, all study participants were asked whether they had begun reconsidering their future profession choices following the VR session. Notably, four students (11%) affirmed that they had reconsidered their later professions. Among these, two students had already determined their future profession prior to the VR session: one student had opted for a non-surgical profession, whereas the other had chosen a surgical profession. Statistical analyses did not reveal any significant differences between the last and non-last year, male and female, or younger (20–25 years) and older (26–30 years) students (Fig. 5C).

### Free-form text feedback regarding VR anatomy teaching

At the end of the survey all study participants were provided the opportunity to offer free-form text feedback and propose future development ideas regarding the VR anatomy teaching. A total of nine students (24%) utilized the free-form text feedback option. One student reported two aspects. The feedback included nine positive comments and suggestions for optimization, which can be summarized as follows: successful introduction of the hard- and software, accurate dimensional representation of the anatomical structures, VR as a complement to the dissection class, fascination of VR-driven future surgical strategies, and implementation of additional VR-based practical aspects. One student criticized the sound system of the VR headsets.

## Discussion

In this study, we demonstrated the feasibility of implementing VR-based surgical mediastinal and abdominal anatomy teaching in medical students, as exemplified by a virtual esophageal cancer patient model. State-of-the-art VR technology enables students to visually observe the complex anatomy of the mediastinum from multiple perspectives. Overall, VR-based surgical topographic anatomy teaching received positive evaluations, and positive student feedback was consistent across training levels, age groups, and different genders. Interestingly, while only approximately half of the students reported a prior VR experience, the majority found the software to be intuitive. Although not statistically significant, our study indicated a potential effect between participation in VR-based teaching and students’ subsequent selection of surgical specialty.

### VR teaching value

Notwithstanding that our study results are based on students’ subjective evaluations using a non-externally validated self-report tool, they align with two recent meta-analyses that examined the effectiveness of VR-based anatomy teaching among medical students and residents. García-Robles et al. showed that extended reality (XR) technologies, including VR applications, generate higher knowledge gains and are rated as more useful than traditional teaching approaches among over 2,000 health science students. Notably, the most significant effect on anatomical knowledge acquisition was observed when these technologies were used in combination with traditional resources as supplementary tools [11]. Similar effects were reported by Wang et al. In their study, 3D visualization technologies for learning human anatomy significantly improved learners’ test scores and satisfaction compared to other methods among 3,000 medical students [16].

The systematic review and meta-analysis published by Moro et al. in 2021, however, revealed no significant effectiveness of VR on anatomical knowledge [17]. This review included eight studies published in the pre-pandemic era (2013–2019) and did not distinguish the technical details and improvements in VR. Due to the increased demand for remote teaching during the COVID-19 pandemic, the e-learning market, including extended reality solutions, has experienced significant growth, with an estimated annual increase of nearly 15% [18]. Consequently, advancements such as higher resolution displays, broader fields of view, lighter headsets, improved controller tracking, and higher fidelity software, such as “Medicalholodeck” for VR, have been introduced over the past five years. Hence, the recent meta-analysis by Salimi et al. demonstrated a significant impact on knowledge acquisition but not on usability [13]. In our study, the entire cohort reported high usability, likely attributable to the aforementioned technical enhancements.

In addition, students receiving a VR-based lecture on esophageal and mediastinal anatomy exhibited a more profound understanding of computed tomography (CT) images and better interpretation of surgical images than those receiving only 3D images. However, spatial recognition scores did not differ between both groups [19]. Although we did not assess spatial recognition, our case-based VR study highlights how complex and challenging mediastinal and esophageal anatomy spanning different anatomical regions may be more readily comprehended through visualization from multiple perspectives.

The medical VR software “Medicalholodeck” has the technical capability to integrate patient-specific 3D radiological imaging data within the metaverse. Although this feature was not applied in the present study, it represents a potential future direction for enhancing anatomical education using real clinical data.

### Instructor’s role during VR session

The overall positive student feedback and self-reported high teaching value may be attributed to the continuous presence of an instructor throughout the teaching session. The instructor not only introduced hard- and software applications but also provided additional relevant anatomical information when students required clarification facilitated by the cloud-based solution. Finally, the esophagectomy procedure was demonstrated. Recently, Ojala et al. showed that the presence of a teacher positively influences learning-related outcomes in VR-based anatomical teaching among medical students [20]. These findings underscore the necessity and beneficial teaching effects of an instructor’s presence. The implementation phase of the VR program should be conducted under the supervision of an experienced instructor, as in the present study. Furthermore, future implementation of VR teaching should include structured ‘train-the-trainer’ programs to ensure consistent, high-quality educational experiences.

Another explanation for the predominantly positive student feedback may be the integration of VR teaching with case-based learning, which has been shown to enhance learning outcomes and motivation for anatomy studies [21].

### Negative side effects of VR teaching

Although students noted technical improvements, such as enhanced audio quality, no cybersickness or additional individual physical or mental limitations were reported. One explanation may be attributed to the utilization of contemporary hard- and software applications and mostly static teaching content, given that several VR studies have documented the occurrence of cybersickness in up to 60–95% of participants [22]. Cybersickness exhibits a negative correlation with anatomy learning and consequently represents a significant adverse effect of VR-based teaching [23].

### Impact of VR teaching of pursuing a future surgical career

Although not statistically significant, our study further revealed a potential influence on students’ future choice of surgical profession, as 11% of all participants stated reconsidering their intended specialization following the VR session. This finding aligns with a randomized trial by Imai et al., who also incorporated VR in esophageal and mediastinal anatomy teaching: students who received a lecture using VR images demonstrated increased interest in surgery [19].

### Limitations and areas for future research

Several limitations of our question-based approach should be noted. First, the study cohort only comprised 37 voluntary participants, limited to only one site in one country, which may introduce a selection bias and potentially fail to represent the broader population of medical students, thus limiting the generalizability of the findings. However, the study cohort was evenly distributed in terms of age, training level, and prior VR experience. The learning success of the anatomical structures was self-rated by the participants through a questionnaire, without additional external validation. No objective, content-based knowledge questions or standardized assessment of anatomical knowledge (e.g. pre- and post-intervention multiple-choice questions) was administered following the VR session, as this study was intentionally designed as a pilot study to primarily assess the implementation, acceptance, and subjective learning experience of a VR-based environment which is a crucial early step in the implementation and adaptation of novel educational technologies.

Due to missing externally validated VR questionnaires for the implementation of VR-based surgical topographic anatomy teaching in medical students, we utilized an amalgamation of two questionnaires which have been used previously [20, 24]. The objective of the new questionnaire was to assess the multiple dimensions of possible benefits, barriers to application, and drawbacks of VR-based surgical topographic anatomy teaching while maintaining brevity. Additionally, the questionnaire indicated a high internal consistency and response rate. No free-form answers indicated dissatisfaction or confusion regarding the questionnaire. Thus, our questionnaire can be considered a pilot for the latter and awaits further validation. Our sample size was insufficient to show or disprove the alignment of the empirical factor structure with the theoretical rationale behind the selection of questions. Future research can use our questionnaire to investigate further aspects of VR-based teaching. Therefore, a larger sample size and an enlarged question pool may be necessary.

Furthermore, students evaluated the overall resemblance of the VR-simulated anatomical structures to their dissection experience without distinguishing between the two software types. Future studies should include separate evaluations of both software applications to better understand how the source of 3D content influences students’ perceptions of anatomical realism. Another important aspect concerns the financial implications of VR-based medical education. Over the past five years, the development of more affordable VR hardware has markedly reduced entry costs – for instance, current devices such as the Meta Quest 3 S are available for approximately €300–350. In contrast, software expenses remain highly variable worldwide, largely depending on functionality, licensing structure, and content depth. According to the cost analysis by Chumbley et al., virtual dissection ranked as the third most affordable modality for anatomy education, following computer-assisted instruction/learning and plastic models reaching up to 400 British Pound per student [25]. Further studies should address cost comparison between digitalized educational materials and conventional teaching methods.

## Conclusion

VR-based instruction for teaching surgical topographic anatomy has not yet been extensively evaluated nor systematically integrated into medical education curricula. Here, we showed that the implementation of VR-based surgical topographic anatomy for the education of medical students is feasible, shows a self-reported teaching value independent of age, gender, or level of education, and may influence subsequent career choices towards a more surgically oriented career path. Although it is unlikely that VR-based anatomy teaching will entirely supplant dissection classes, VR will undoubtedly be incorporated into the curriculum, in combination with traditional resources. Furthermore, the virtual anatomy experience could serve as a potential complement or substitute for cadaveric dissection in settings where cadaveric donors are unavailable.

Therefore, it is important to further elucidate the differences between VR, dissection classes, and other traditional teaching methods and to determine how to optimally integrate these approaches to achieve maximum efficacy in the future. In this context, time-cost efficiency calculations will also play a major role, as VR teaching applications offer considerable flexibility in terms of temporal and spatial constraints as well as the potential for repetitive training.

## Supplementary Information

Supplementary Material 1.

Supplementary Material 2.

Supplementary Material 3.

## Data Availability

The datasets used and/or analyzed during the current study are available from the corresponding author on reasonable request.

## Abbreviations

VR: Virtual reality
2D: Two-dimensional
3D: Three-dimensional
XR: Extended reality
CT: Computed tomography
