# Supplementary material for: Implementing virtual reality based surgical topographic anatomy for the education of medical students: a pilot study
Source: BMC Med Educ. 2026 Jan 9;26:151. doi: 10.1186/s12909-025-08563-z (PMC12849294; doi:10.1186/s12909-025-08563-z)
Supplement: Supplementary file 1 — Supplementary Material 1. [file 12909_2025_8563_MOESM1_ESM.docx]

**Questionnaire**

**1. Student: Male Female Other**

**Age (years): Handedness: Right Left**

**2. Prior VR experience: Yes No**

**3. Do you already know your later profession: Yes No**

**4. If yes: Surgical profession Non-surgical profession**

**5. General questions of the VR exercises:**

Usefulness 0-10 (0=useless, 10=very useful):

Usability 0-10 (0=difficult to use, 10=very workable):

Did you learn something new 0-10 (0=nothing, 10=very much):

**6. VR added value to teaching,** 0-10 (0=none, 10=very much)**:**

**7. The exercise fit physically, mentally, and educationally for me**:

**Yes No**

**8. Do the VR anatomical structures appear to resemble the properties of human tissue compared with your dissection class?**

**Yes No**

**9. Do you think there is a teaching advantage in using the VR phantom versus a live patient?**

**Yes No**

**10. Did the instructor adequately demonstrate how to use the VR technique?**

**Yes No**

**11. Did you start rethinking about your later profession after this VR session?**

**Yes No**

**12. Free-form text feedback:**

*Adapted and modified from Ojala et al., 2022 and Stone et al., 2022.*
