# Supplementary material for: Implementing virtual reality based surgical topographic anatomy for the education of medical students: a pilot study
Source: BMC Med Educ. 2026 Jan 9;26:151. doi: 10.1186/s12909-025-08563-z (PMC12849294; doi:10.1186/s12909-025-08563-z)
Supplement: Supplementary file 2 — Supplementary Material 2. [file 12909_2025_8563_MOESM2_ESM.docx]

**Supplementary Figure 2. Factor Analysis of the questionnaire**

A factor analysis was conducted using all non-demographic questions, except for two questions: the role of the instructor and personal physical and mental views of the VR exercises, as these were answered unanimously (except for one response). Supplementary Figure 2 shows a scree plot of the factors and principal components against the simulated (resampled) data. This suggests a two-factor structure that is consistent with the $\chi^{2}$ goodness-of-fit tests for one factor ($\chi^{2}$=29.72, df=14, p<0.01) and two factors ($\chi^{2}$=5.96, df=8, p=0.65).


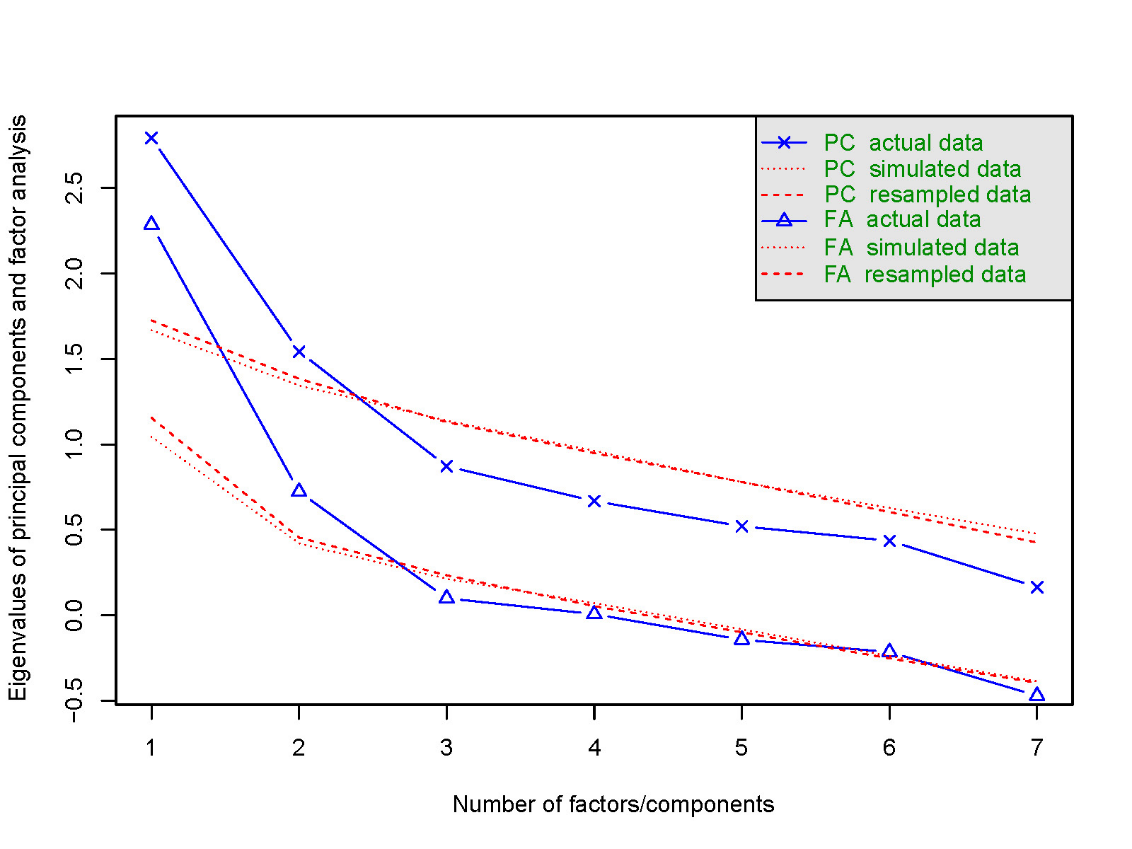


**Supplementary Figure 2.** Scree plot of factors and principal components against simulated (resampled) data. PC: principal component, FA: factor analysis.

The two-factor solution factor loadings of the varimax rotation are summarized in Supplementary Table 1.
