# Supplementary material for: Implementing virtual reality based surgical topographic anatomy for the education of medical students: a pilot study
Source: BMC Med Educ. 2026 Jan 9;26:151. doi: 10.1186/s12909-025-08563-z (PMC12849294; doi:10.1186/s12909-025-08563-z)
Supplement: Supplementary file 3 — Supplementary Material 3. [file 12909_2025_8563_MOESM3_ESM.docx]

**Supplementary Table 1.** Two-factor solution factor loadings of varimax rotations.

| **Question** | **Factor 1** | **Factor 2** |
| --- | --- | --- |
| Usefulness of VR exercises | 0.670 | 0.584 |
| Knowledge gain through VR exercises | 0.838 | 0 |
| General teaching value of VR exercises | 0.628 | 0.194 |
| Usability of VR exercises | 0.601 | 0 |
| Resemblance of the VR-simulated anatomical structures to dissection class | -0.133 | 0.976 |
| Teaching advantage of a virtual patient compared with a live patient | 0.124 | 0.402 |
| Rethinking of later choice of profession after the VR session | 0.342 | 0.109 |
